# In This Issue: Volume 116, Issue 2, February 2025

**DOI:** 10.1111/cas.16457

**Published:** 2025-02-01

## Current status and future direction of cancer research using artificial intelligence for clinical application

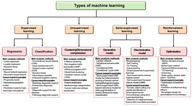

Artificial intelligence (AI) is transforming many sectors, including healthcare, where it is making significant strides in cancer research and treatment. Recent breakthroughs, such as advanced AI tools like ChatGPT and other deep learning technologies, have opened new possibilities for detecting, studying, and treating cancer more effectively.

AI has shown great promise in analyzing medical images like X‐rays, MRIs, and biopsies, helping doctors detect cancer earlier and more accurately than ever before. Beyond imaging, AI is also being used to analyze complex datasets such as DNA profiles and electronic medical records. These insights enable the development of personalized cancer treatments tailored to each patient's unique genetic and clinical profile. Such targeted approaches are already improving outcomes for many patients by offering more precise and effective treatment options.

However, integrating AI into cancer care is not without challenges. While AI often performs well in controlled tests, its reliability in real‐world clinical settings can vary. The complexity of AI systems makes it difficult for doctors to fully understand or trust its recommendations. Differences in medical practices and equipment across hospitals can also affect AI's performance. Moreover, AI systems, like any tool, are not perfect—they can make mistakes or generate misleading results, posing potential risks to patients.

To address these issues, researchers and regulators are taking a cautious approach. Rigorous clinical testing is essential to ensure AI tools are both safe and effective. Transparency in how AI reaches its conclusions is critical for building trust among healthcare professionals. At the same time, protecting sensitive patient data remains a top priority.

Progress in this field requires collaboration across disciplines. Experts in medicine, bioethics, sociology, law, and patient advocacy are working together to ensure AI benefits not just researchers but also patients and society as a whole. The focus is on long‐term impact, emphasizing patient safety and public well‐being over hasty implementation.

AI holds enormous potential to transform cancer research and treatment. By combining advanced technology with a careful, collaborative approach, researchers aim to harness AI's power responsibly, paving the way for more precise, effective, and equitable cancer care in the future.

https://onlinelibrary.wiley.com/doi/10.1111/cas.16395

## Rewired chromatin structure and epigenetic gene dysregulation during HTLV‐1 infection to leukemogenesis

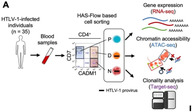

Adult T‐cell leukemia‐lymphoma (ATL) is an aggressive cancer caused by the human T‐cell leukemia virus type 1 (HTLV‐1). This disease primarily affects T‐cells, a critical component of the immune system, and can manifest as a lymphoid malignancy with leukemic change. ATL has a very poor prognosis, with many patients surviving less than a year after diagnosis. HTLV‐1 spreads through unprotected sex, breastfeeding, unsafe blood transfusions, or non‐sterile needles, highlighting the need for preventive measures and better treatment options.

ATL develops when HTLV‐1 alters normal gene activity in T‐cells, turning them cancerous. This study investigated how the virus reshapes the genetic and structural framework of infected cells, even before cancer develops. Researchers used advanced techniques, including RNA sequencing (RNA‐seq) to analyze gene activity and transposase‐accessible chromatin sequencing (ATAC‐seq) to study how DNA is packed and accessed by cellular machinery.

The results revealed that HTLV‐1 begins altering T‐cells at early stages of infection, well before the onset of ATL. Over 60% of the gene activity changes in infected cells were strikingly similar to those observed in full‐blown ATL cells, suggesting that the groundwork for cancer is laid early. Genes involved in cancer progression, such as *CADM1*, *CCR4*, *IL2RA*, and *EZH2*, were found to be more active, while immune‐regulating genes like *CD7* and *CD26* were less active.

Moreover, the study uncovered large‐scale changes in how DNA is packaged in the nucleus. DNA regions that became “open” were more accessible to transcription machinery and exhibited increased gene activity, while “closed” regions showed reduced activity. Specifically, 2252 DNA regions became more open, and 14,527 regions became closed in infected cells, demonstrating HTLV‐1's significant impact on chromatin structure.

The viral protein Tax emerged as a key driver of these changes. Tax interacts directly with DNA and host cellular machinery, altering chromatin structure and gene expression. One critical gene affected by Tax is *RASGRP3*, which is usually inactive in healthy T‐cells. In infected cells, RASGRP3 became highly active, triggering a signaling cascade through the ERK pathway that promotes uncontrolled T‐cell growth. When researchers blocked *RASGRP3* activity, T‐cell growth was significantly reduced, making this pathway a promising target for future therapeutic interventions.

This study provides valuable insights into how *HTLV‐1* induces early and widespread changes in DNA structure and gene activity, setting the stage for ATL development. These findings underscore the potential for developing targeted therapies that could intervene in the early stages of infection to prevent cancer or treat it more effectively. By revealing specific genes and pathways involved in ATL progression, such as the RASGRP3‐ERK pathway, this research offers a foundation for identifying novel drug targets to combat this aggressive disease.

https://onlinelibrary.wiley.com/doi/10.1111/cas.16388

## Phospholipase D2 downregulates interleukin‐1β secretion from tumor‐associated macrophages to suppress bladder cancer progression

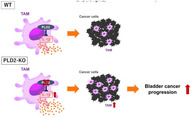

Bladder cancer (BC) is one of the leading causes of cancer‐related mortality worldwide. Its progression often involves metastasis to the muscles and frequent recurrence, even after successful treatment. Despite the development of immunotherapy, the prognosis for metastatic BC remains poor, largely due to the tumor microenvironment (TME). This TME, a complex network of cells and molecules surrounding the tumor, is known to contain fewer immune cells than other cancers, contributing to treatment resistance. Identifying factors that influence the TME may open new avenues to enhance BC treatment responses.

Phospholipase D (PLD) isoforms, PLD1 and PLD2, are enzymes involved in molecular signaling pathways across various cancers. Although PLDs generally promote tumor growth and metastasis, their specific roles in BC and its TME are not fully understood. While prior research has highlighted PLD1's contribution to tumor metastasis within BC cells, the role of PLD2, particularly in the TME, had remained unclear.

In this study, researchers investigated the impact of PLD2 on BC progression and the TME using a mouse model with the *Pld2* gene completely inactivated (Pld2‐KO mice). Transcriptomic analyses and gene expression studies revealed that PLD2 plays a suppressive role in BC progression. Its absence led to increased cancer invasiveness, indicating that PLD2 is involved in limiting tumor growth via immunosuppressive pathways in the TME.

One key finding of this study was the significant increase in tumor‐associated macrophages (TAMs) in the TME of Pld2‐KO mice. TAMs, immune cells that interact directly with tumor cells, were found to promote BC survival and correlate with poor prognosis. Further investigations revealed that PLD2 suppresses the production of interleukin‐1β (IL‐1β), a cytokine that drives TAM proliferation. Without PLD2, TAMs produce elevated levels of IL‐1β, resulting in greater TAM proliferation and enhanced BC progression.

The study concluded that PLD2 is a critical gatekeeper in the battle against bladder cancer, curbing tumor progression by taming TAMs and limiting IL‐1β production. By targeting PLD2, researchers could potentially rewire the TME, paving the way for more effective immunotherapy strategies and offering new hope for patients battling this challenging disease.

https://onlinelibrary.wiley.com/doi/10.1111/cas.16393

